# Employing General Linguistic Knowledge in Incidental Acquisition of Grammatical Properties of New L1 and L2 Lexical Representations: Toward Reducing Fuzziness in the Initial Ontogenetic Stage

**DOI:** 10.3389/fpsyg.2021.768362

**Published:** 2022-01-31

**Authors:** Denisa Bordag, Andreas Opitz

**Affiliations:** ^1^Herder Institute, Leipzig University, Leipzig, Germany; ^2^University of Haifa, Haifa, Israel

**Keywords:** mental lexicon, conversion, second language acquisition, fuzzy representation, incidental acquisition, word categories (parts-of-speech)

## Abstract

The study explores the degree to which readers can use their previous linguistic knowledge, which goes beyond the immediate evidence in the input, to create mental representations of new words and how the employment of this knowledge may reduce the fuzziness of the new representations. Using self-paced reading, initial representations of novel identical forms with different grammatical functions were compared in native German speakers and advanced L2 German learners with L1 Czech. The results reveal that although both groups can employ general knowledge about German grammar when establishing new representations, the L1 native speakers outperform the L2 learners: Their new representations have more precise structure and are better differentiated from related representations with respect to their grammatical information. Modeling consequences of these findings are discussed in the context of the Ontogenesis Model of the L2 Lexical Representation and the Fuzzy Lexical Representation Hypothesis.

## Introduction

In recent years, the properties of newly acquired lexical representations have gained more attention compared to those that are well established and frequently used. The focus has been primarily on the acquisition of meaning and its integration in the semantic network (for L1 e.g., Perfetti et al., 2005; Breitenstein et al., 2007; Clay et al., 2007; Mestres-Missé et al., 2007; Borovsky et al., 2010; Tamminen and Gaskell, 2013; for L2 e.g., Elgort, 2011; Bordag et al., 2015a,2017a,^2018^) and on the equivalent questions regarding word form (for L1 e.g., Shtyrov et al., 2010; for L2, e.g., Bordag et al., 2017b). However, there is basically no research exploring the initial representations of grammatical features in natural languages, in particular in the incidental acquisition scenarios when they need to be inferred from the linguistic context. Studies related to such topics usually have a different focus or background. For example, several studies investigate grammar acquisition in incidental learning, but their primary concern is not to explore the mental representations of the newly established grammatical features, but usually rather to assess learning gains under different reading conditions. Such studies address either effects of reading on overall grammar competence (i.e., not focusing on a particular grammar feature, e.g., Elley and Mangubhai, 1983) or a single grammatical feature, but are not concerned with its mental representation or how it interacts with previously acquired grammatical knowledge as is the case with our study. As an example, Aka (2020) explores the efficiency with which Japanese learners of English acquire to-infinitives used as nouns during reading while varying the amount of the target features in the input. Other authors, such as Shintani and Ellis (2010) or Song and Sardegna (2014) address incidental grammar acquisition of individual grammatical features (prepositions and -s plurals, respectively) during reading within a similar framework. Outside the area of research on reading, grammar acquisition is explored in studies with novice learners [e.g., noun-adjective agreement in Russian in Denhovska et al. (2016); plural -s and copula be in Shintani (2015)] and in artificial grammar learning (e.g., Grey et al., 2014; Monaghan et al., 2021; Rebuschat et al., 2021). In both cases, effects of previously acquired grammatical knowledge on the acquisition of features of the target grammar are not in focus and neither is the mental representation of the acquired features.

Similarly, the first versions of the most recent frameworks and approaches, such as the Ontogenesis Model of the L2 Lexical Representation (OM; Bordag et al., 2021a) and the related Fuzzy Lexical Representation (FLR) Hypothesis (Cook and Gor, 2015; Gor and Cook, 2020; Gor et al., 2021) that address the development of individual lexical representations and their quality only marginally touch upon the grammatical aspects. The OM addresses the development of lexical representations along three dimensions: the dimension of linguistic domains, the dimension of mappings between domains, and the dimension of networks of lexical representations. The dimension of linguistic domains that constitute a lexical entry has several sub-domains. The model focuses on the phonological, orthographic, and semantic domains as they comprise information which is stored at the lexical entry according to a general consensus. With respect to grammar, the situation is more complex, which is one of the reasons why it has not been addressed in the model blueprint. While some aspects of grammar such as agreement or word order are assumed to be handled on the processing level, other aspects, in particular morphosyntactic features, are assumed to be stored at the lexical entry. For the processing level of grammar, there is, to our knowledge, no model or approach that would operate with the concept of fuzziness. However, there are approaches that address topics that could be related to fuzziness in a broader sense such as the Shallow Structure Hypothesis (e.g., Clahsen and Felser, 2006, 2017). According to this hypothesis, L2 learners dispose of the same processing architecture and mental-processing mechanisms as L1 speakers, but they have “problems building or manipulating abstract syntactic representations in real time” (Clahsen and Felser, 2017, p. 2) and underuse syntactic information in online processing. Consequently, their processing could be seen as “fuzzier.” However, it remains to be explored to which degree the concept of fuzziness would need to be adapted to suite also processing models in which fuzziness is seen more as a binary property.^1^ With respect to the representational level that we address in our study, grammatical features at the lexical entry subsume both the so-called internal grammatical features with fixed values that need to be acquired (e.g., word class, grammatical gender, or declension class of nouns, number of singularia and pluralia tantum, subcategorisation frame, conjugational class or aspect of verbs, and declension class of adjectives), and the so-called external features with variable values that need to be set anew during processing each time (e.g., number, case, tense, grammatical voice, and gender of adjectives) (Bordag and Pechmann, 2009). For models supposing the existence of a so-called lemma as a component of a lexical entry (in addition to, e.g., a phonological form, earlier ‘lexeme’), such as the Interactive Activation Model (Dell, 1986) or the Levelt Model (Levelt, 1989; Levelt et al., 1999), the lemma is supposed to be where such morphosyntactic features are stored [but cf., e.g., the Independent Network Model of Caramazza (1997) that dispenses with the notion of a lemma]. The part of grammar with a representational character and that is represented at the lexical entry would be a candidate for the grammar/morphosyntactic domain in the OM model.

The concept of fuzziness plays an important role in the OM model and is further developed in the FLR hypothesis (in particular Gor et al., 2021) that shares its focus on the quality of lexical encoding with the lexical quality hypothesis developed for L1 reading (Perfetti and Hart, 2002; Perfetti, 2007). Lexical representations undergo a developmental change during which the degree of fuzziness decreases untill a target stage is reached. This target stage of a lexical representation’s ontogenesis, for which fuzziness is reduced to zero, is called ‘optimum.’ Fuzzy lexical representations are described in the FLR hypothesis as having imprecise, low-resolution or fuzzy encoding of their form and/or meaning, and potentially also the mapping between them. Their less distinct boundaries result in their reduced differentiation from neighboring representations.

The OM assumes that the development of a lexical representation can follow various scenarios depending on multiple factors such as the learning conditions or the current state of the learner’s mental lexicon. As an example, the authors describe several possible developmental curves for the acquisition of the semantics of the word *dandelion* (for more details please cf. Bordag et al., 2021a, pp. 9–10 and Figure 5). In the simplest scenario, the word form is directly linked to an already existing semantic representation (possibly via the L1 form for novice learners, De Groot et al., 1994; see Bordag et al., 2017a for a detailed description), as it is typically the case when L1–L2 vocabulary pairs are learned, and the translation equivalency is given. In this case, there is a sudden rise of the semantic ontogenetic curve toward the optimum. In more complex scenarios, the equivalency may need to be discovered in a cumulative way and initially only highly fuzzy semantic representations emerge that consist of, for instance, only very general features (e.g., ‘a kind of blossoming flower’), or that comprise a specific but incomplete set of features. Over time, such representations may get more precise and semantically richer. This is typically the case when the meaning needs to be inferred in incidental vocabulary acquisition and depends on the input quality with respect to the available cues (Ellis and Collins, 2009). In such a case, fuzziness is reduced more gradually and the rise toward the optimum is less steep and/or may proceed in jumps.

Fuzziness is primarily viewed as a property of less familiar words (i.e., whose representations are lower on the ontogenetic curve that culminates in the optimum) in both L1 and L2. However, since less familiar words are more numerous in the L2 mental lexicon and because L2 learners experience more difficulties with encoding the phonological form and meaning of L2 words, establishing strong mappings between them, and integrating new L2 lexical entries in the lexical network, fuzziness is more pervasive in the L2 compared to L1^2^. Though the OM explicitly and the FLR hypothesis implicitly assume that fuzziness also affects grammatical encoding, the topic is not developed in either approach. As we will show in the current study, the basic concepts of these approaches such as fuzziness or optimum can also help to understand the characteristics and the initial development of new lexical representations at the grammatical level.

In our study, we ask to what degree readers or listeners can use their previous linguistic knowledge, which goes beyond the immediate evidence in the input, to create the mental representation of a new word and how the employment of this knowledge may reduce the fuzziness of the new representations. We were particularly interested in whether new mental representations are idiosyncratic in that they contain only grammatical information that could be derived directly from the linguistic context in which the new word appeared, or whether their establishment is assisted also by the information anchored in the reader’s general knowledge about the grammar of the language, and if yes, how the engagement of this knowledge interacts with the fuzziness of the representation.

We found an empirical domain suitable for addressing this question in the area of German morphology. In German, every verb can be turned into a noun via a morphological process called conversion or zero-derivation. The crucial point of that process is that it operates without overt affixation (hence the name ‘zero-derivation’): The product, the conversion noun, is formally identical to (some) morphological forms of the base verb. Thus, a German infinitive form like SPIELEN (‘to play’) can be converted into a form-identical conversion noun ‘das SPIELEN’ (‘the playing’). This process is highly productive, and any German verb can be turned into an uncountable neuter (with respect to gender) noun this way. Though the mental representation of conversion nouns is still controversial, the most recent research supports the hypothesis that German deverbal conversion nouns are nested as word-category-specific subentries under a basic lexical entry that comprises also a subentry for a verbal representation. In their priming experiments with grammaticality judgments, Bordag and Opitz (2021) and Opitz and Bordag (2021) compare priming between formally identical primes and targets while manipulating the function of the primes, one of which being that of conversion [e.g., prime: *das – SPIELEN* (‘the playing’), target: *wir – SPIELEN* (‘we play’)^3^ ]. The comparison of the priming effects in the different prime conditions (identical, inflected, infinitive, conversion noun, and inflected countable noun) allowed the authors to assess whether different, partially different, or the same representations were accessed. The priming patterns suggest the existence of complex lexical entries where the upper level is word-class neutral, and the lower levels (subentries) are specified for word classes and word-class-specific information (verbal sub-entry for verbal forms and nominal subentry for conversion nouns).

One of the questions we ask in our study is whether readers, who encounter a particular word form in a text such as an inflected verb form in 3rd person plural, establish only a simple lexical entry comprising only the given, i.e., verbal, information, or whether they can establish a more complex lexical entry also containing the conversion noun subentry based on linguistic generalization (for which they do not find any cue in the immediate linguistic context, though); and vice versa: Does the presentation of a new word as a conversion noun lead to establishing a simple nominal representation or does the new word’s lexical entry also contain the verbal component?

Previous research on how existing general linguistic knowledge can affect representation of new linguistic information is scarce and emerges rather as a by-product in studies addressing other aspects of grammar acquisition. Bordag et al. (2015b) explored incidental acquisition of grammatical features of verbs during reading. They focused on subcategorisation and the (ir)regularity status of verbs. These two verb properties differ in that while a dominant, more frequent category can be determined for (ir)regularity status (namely the regular conjugation), no such generalization is possible for subcategorisation – a verb can be transitive or intransitive with basically the same chance. In their experiments, native and non-native speakers of German read short texts followed by several sentences that participants had to read in a self-paced manner. The introductory texts contained a conjugated novel verb repeated three times, whose meaning participants could derive from the context. The verb was then repeated in one of the self-paced sentences. In the congruent condition, the properties of the verb complied with its properties in the introductory text [e.g., the same subcategorisation frame or the same conjugation type (regular vs. irregular)]. In the incongruent condition, one of the two properties was violated, e.g., the verb was presented in a different subcategorisation frame than in the introductory text or it was presented as regular while conjugated as irregular in the introductory text or vice versa.

Bordag et al. (2015b) found that both native and advanced non-native readers could derive and store the information about the subcategorisation frame of a novel verb after just three occurrences in a text. However, contrary to the L2 learners, the L1 participants seemed indifferent to the (ir)regularity status of the novel verbs as it was presented in the introductory texts: No matter whether the verb was conjugated regularly or irregularly, the irregular conjugation was always perceived as a violation in the self-paced reading test phase. The authors interpret the finding through a “learning by unlearning effect”: in their long experience with their native language, the L1 readers learned that regular conjugation is productive and that the set of irregular verbs is a rather small, closed group of verbs and they are certain to know all its members. Having learned this, they cease to acquire information about conjugation type from input and instead assume – based on their general knowledge about the language – that all new verbs are regular. If an unknown irregular form appears, they consider it implausible, irrespective of the evidence in the input, so the actual evidence in the particular context is overridden by the general knowledge. These findings indicate that – where applicable – L1 participants not only draw generalizations about linguistic properties and categories, but that these generalizations can also drive the acquisition and affect the setting of properties in newly established representations. On the other hand, L2 learners seem to be more driven by the actual input when acquiring linguistic properties of new words and less able to employ general knowledge about the language (cf. also the stronger focus on verbatim information in L2, e.g., Sampaio and Konopka, 2012; Bordag et al., 2021b; and the L2 form prominence, e.g., Jiang and Zhang, 2019). How the involvement of general knowledge may interact with the degree of fuzziness of the newly established representation, which is one of the aims of this study, has been neither directly explored nor actually addressed thus far.

## The Present Study

In the present study we applied a method similar to Bordag et al. (2015b) to test whether participants can use general knowledge about the acquired language to establish complex lexical entries that contain information which goes beyond the immediate evidence in the text. More specifically, we asked whether an encounter with each of the forms (verb and conversion noun) triggers the establishment of a new lexical entry that also contains the representation of the other one, or whether for instance, only the encounter with the more basic form (from which the other form is derived, i.e., presumably the verb) enables the establishment of a complex representation containing also the specifications for the derived functions (i.e., the de-verbal, converted noun form).

In addition, we wanted to explore whether the employment of generalized grammatical information differs for native speakers and advanced learners of German and how this may be related to the higher degree of fuzziness observed for L2 representations compared to L1 so far, primarily in the domains of phonology and semantics (Bordag et al., 2021a). As previous research indicates, L2 learners might have a limited ability to engage this knowledge (as it is also typically at a lower level of acquisition compared to L1) and may thus be more dependent on the verbatim, word-form-related information in the input in general and when establishing new lexical entries in particular. To our knowledge, no previous research targeting a direct comparison between adult L1 and L2 acquisition in this area has been reported.

In the experiments in this study, participants read short German texts that contain a novel, previously unknown lexical item repeated twice. Each text is followed by a sentence read in a self-paced reading manner that includes the critical item either as an inflected verb form, an infinitive verb form, a conversion form, or a countable noun form. All forms shared the same stem and the ending –*en* that had a different function for each form. Thus, the target forms in the SPR sentences were all formally identical and differentiated only through the slightly different syntactic context of the sentence part which preceded them. This way we could compare reading times of form-identical words that differed only in their grammatical specifications (verbal forms, conversion nouns, and countable nouns) that were either present in the previous input (i.e., in the short texts), or not. In addition, using this version of the self-paced reading task enables to test the acquired knowledge for every single new word directly after it had occurred in input for the first time (compared to, for example, priming experiments).

The countable noun condition was included to serve as a kind of control condition. The countable noun (e.g., die MIETEN ‘the rents’) is homonymous with the other forms [e.g., MIETEN can also mean *they rent, we rent, to rent*, (*the*) *renting*], but its derivation is not a productive process in German: Not all German verbs have such derivations (their number is rather very limited) and neither their base forms (die Miete – ‘the rent’ in nominative singular), nor their meaning or grammatical gender are predictable from the verb stem from which they are derived historically. Previous research showed that homonymous countable nouns are represented as separate lexical entries (Bordag and Opitz, 2021; Opitz and Bordag, 2021). Therefore, if participants establish lexical entries that are precise and thus distinctly differentiable from other representations, they should not process the countable noun in the SPR sentence as the recently established verb/conversion noun representation, but rather respond to it as a new representation encountered for the first time (alternatively: respond to it as a violation). In this case, we should expect longer reading times for this control condition than for the other conditions. Contrary to the existence of the verb and the corresponding conversion noun that mutually condition themselves, the existence of the countable noun entry cannot be extrapolated from the more general, productive rules of the German language. Crucially, this control condition shared with the other experimental conditions the fact that the critical word was formally identical. Thus, any differences in reading times could not be caused by differences in form overlap.

Participants were tested in two experimental versions, A and B. The two versions differed in the function of the novel word which appeared in the short preceding texts: In version A, the new word was presented as a conjugated verb form; in version B it was a conversion noun form.

We hypothesized that if readers employ more general linguistic knowledge about the German language system when establishing a new representation, the resulting representations would be different to those if readers establish the representation relying solely on the information available in the immediate input. Since all German verbs can be converted into conversion nouns, readers could establish a complex lexical entry also containing the conversion noun grammatical information when encountering an inflected verb form or containing also the verbal grammatical information when encountering a conversion noun form based on their previous grammatical knowledge. In this case, we would expect the same reading times in both SPR verbal conditions (inflected and infinitive) and in the conversion condition, because in all cases participants would be accessing an already established entry containing full grammatical information (Hypothesis 1). However, if participants could not access more general linguistic knowledge (“to every verb there is a conversion noun” or “to every conversion noun, there is a verb”), they would only be able to establish a simpler entry containing only the grammatical information (verbal or conversion noun) that appeared in the text. In this case, we would expect longer reading times in the SPR condition that contains the form that did not appear in the initial text (Hypothesis 2). The longer reading times would either arise because that (part of the) representation could not be established yet based on the previous input and may become established only during reading of the SPR sentence in which it appears for the first time, or because this first-time-occurring form (in the SPR sentence) would be perceived as a violation because its word class is incongruent with the information readers induced and represented based on the previous text input (along the same argumentation as presented for the countable noun condition above).

By employing versions A (verbal form in text) and B (conversion noun in text), we want to explore whether readers’ ability to use more general linguistic knowledge for acquisition and thus to establish complex lexical entries is dependent on or modulated by the grammatical type of input. We stipulated that participants will be either able to establish a more complex lexical entry comprising both the verbal and the conversion noun information irrespective of whether a verbal form or a conversion noun appears in the input (Hypothesis 1A), or that their ability to employ general grammatical knowledge will be limited or otherwise modulated when one of the forms (verbal or conversion noun) appears in the text input (Hypothesis 1B). For example, the fact that for every verb there is a conversion noun might be easier to generalize and employ in acquisition than that for every conversion noun there is a verb. These differences or asymmetries could be related to the fact that e.g., the higher frequency of the verbal forms compared to the conversion noun forms, conversion nouns are derived from verbs and thus more specific, or – in the case of the L2 learners – in language instruction the typical information shared in the classroom is that one can make a noun from every verb by using the neutral article *das* (formulation of a one-directional rule).

With respect to the differences between the two populations, we expect L1 speakers to be better at using their general linguistic knowledge for acquisition than the L2 learners (cf. also Bordag et al., 2015b) and that the L2 representations may manifest greater fuzziness than the L1 representations. However, since our L2 learners are very advanced, they might already possess the same abilities in this respect as the L1 speakers despite the explored linguistic phenomena not having equivalents in their native language. No similar homonymy of forms with corresponding functions exists in Czech, however, the concept of conversion is familiar to Czech native speakers as it exists, for example, between adjectives and nouns. The explored type of conversion in German is structurally very easy, completely regular, and very productive. It thus enables L2 learners to enlarge their competence significantly at very low costs. As such, conversion is typically learnt and mastered already rather early in L2 German, at the latest at the B1 level (at least for the Czech learners). Its formation in German is significantly easier than the formation of the Czech derived noun that corresponds in its function to the German conversion noun (in German: *sprechen* – *das Sprechen*, *mieten* – *das Mieten*, in Czech: *mluvit* – *mluvení*, *pronajmout* – *pronajmutí*). It can be thus safely expected that Czech learners at B2/C1 level are well familiar with the phenomenon.

Based on previous research, we also expect that critical effects may appear at the spill-over region in addition to the novel word itself. This is in line with Reichle et al. (2009) model of eye-movement control called “E-Z Reader 10,” according to which processing difficulty can occur either at the lexical or post-lexical processing stage. The lexical processing stage comprises a word-familiarity check and lexical access, while the higher-order post-lexical processing involves the integration of the currently fixated word *n* “into the higher-level representations that readers construct online” (Reichle et al., 2009, p. 5). Given that the word form is the same in all our conditions, we can stipulate that it can pass the word-familiarity check without differences related to the different functions of the critical word. However, internal properties of the new representation are relevant for both lexical access and integration of the critical word into higher-level representations. Therefore, we also analyze the spill-over region, in which a word-class mismatch or grammatical properties mismatch (countable noun vs. non-countable conversion noun) between the novel item in the introduction text and the SPR sentence might play a stronger role due to difficulties in integrating a word with an unexpected word class or grammatical properties into the sentence context.

We first present the results of both experiments for the L1 and then for the L2 group. We decided on this order of presentation because our primary question is whether generalized linguistic knowledge is employed during establishment of new lexical entries. We assume that it is more likely to find evidence for it with adult native language speakers, which is why we address this group first. In the second step, we address the same question for advanced L2 learners to explore whether the L2 acquisition procedures work the same as in adult L1. In addition, we examine the patterns of results of both groups to explore whether there are indications of fuzziness in the initially established representations, which we expect especially in L2. Finally, we present an overall analysis of all four experiments that directly compares the L1 and the L2 data and confirms the patterns observed in the language separate analyses.

## Native Participants: Experiments L1A and L1B

In both experiments, participants read short texts in which a novel word (pseudoword) was introduced. After each text, participants read sentences in a self-paced reading manner. In some of the sentences the novel word appeared again, but partially in a different grammatical form.

Methods and procedures for both experiments were mostly identical, except for the grammatical form of the novel word introduced in the text. In Experiment L1A, the novel word was introduced as an inflected verb; in Experiment L1B, it was introduced as a conversion noun.

In the following we report all methods for Experiment L1A and L1B together, highlighting the aspects in which the experiments differed.

### Methods

#### Participants

In Experiment L1A, 72 native speakers (56 female and 16 male) were tested with a mean age of 26.9 years (*sd* = 7.90, range = 18–56). Most participants were university students.

In Experiment L1B, a total of 70 native speakers (48 female and 22 male) were tested with a mean age of 28.9 years (*sd* = 6.9, range = 18–56). None participated in Experiment L1A. Most participants were university students.

#### Materials

##### Items

Twenty-four concrete German verbs with a very low frequency were selected that were mostly unknown to L2 learners at B2 to C1 level as assessed in a pre-test. These verbs were later replaced by pseudoverbs to guarantee that the critical words in the study were completely unknown to all participants (e.g., *gaffen* ‘to gawp’ was replaced by pseudoverb *brössen*). The pseudoverbs were constructed using the computer program Wuggy (Keuleers and Brysbaert, 2010) and followed German orthography and phonotactics (see Hulstijn, 1992). Care was taken that they did not resemble existing words in other languages, in particular in Czech and English. Table 1 lists all novel verbs used in the experiment with their corresponding low-frequency counterparts.

**TABLE 1:** List of items.

Low-frequency word	English translation	Novel word (pseudoword)
Schnitzen	‘To carve’	Fienen
Trödeln	‘To dawdle’	Belfen
Roden	‘To uproot’	Paufen
Gaffen	‘To gawk’	Brössen
Flanieren	‘To stroll’	Jollen
Flattern	‘To flutter’	Tinfen
Plaudern	‘To twaddle’	Zöcheln
Gröhlen	‘To bawl’	Jühnen
Hausieren	‘To peddle’	Rahnen
Kippeln	‘To tipple’	Döcheln
Lispel	‘To lisp’	Plimmen
Nisten	‘To nest’	Wucken
Gurgeln	‘To gurgle’	Zwaulen
Flunkern	‘To fib’	Meifen
Keimen	‘To germinate’	Hunken
Haaren	‘To shed (hair)’	Kleupen
Dösen	‘To doze’	Nieben
Schnurren	‘To purr’	Elmen
Modern	‘To molder’	Lörren
Schielen	‘To squint’	Gäpfen
Brodeln	‘To seethe’	Sülfen
Schlüpfen	‘To hatch’	Fähsen
Rascheln	‘To rustle’	Alzen
Schweißen	‘To weld’	Schünen

##### Texts

Twenty-four short texts were constructed in such a way that the meaning of the 24 verbs could be inferred from them. They comprised 3–5 sentences. The low-frequency verb itself was replaced by a pseudoword. Each pseudoword appeared in its corresponding text twice.

In Experiment L1A, the pseudoword appeared both times as an inflected verb form: Once in 3rd person singular in present tense (e.g., *er brösst*, meaning ‘he gawks’), and once inflected 3rd person plural (e.g., *viele Leute brössen*, meaning ‘many people gawk’).

In Experiment L1B, the pseudoword appeared as a nominalized form (a conversion noun) that was presented twice in the text, once with the article ‘das’ (e.g., *für das Brössen*, meaning ‘for the gawking’) and once in genitive with the article ‘des’ and genitive inflection on the noun (e.g., *wegen des Brössens*, meaning ‘because of the gawking’).

The final selection of the 24 texts was a result of a sequence of two pre-tests, in which all novel words were replaced by a dummy word *xarren*/*Xarren*. Participants were instructed to guess the meaning of the dummy word for each text and rate on a six-point scale how confident they were regarding their guess and how easy it was to deduce the meaning. Additionally, they rated the readability of each text and could leave additional comments regarding each text. In the first pre-test, 36 candidate texts were rated by native speakers (*N* = 48). The texts were presented in two versions, once with the dummy word in the function of an inflected verb (*xarren*), once in a function of a conversion noun (*Xarren*). For each participant, half of the texts appeared with the dummy word in one function and the second half in the other function. Before the second pre-test, the texts were optimized and submitted to another group of native speakers for rating. The 24 texts that scored best in the second pre-test were chosen as final text items for the experiment. The summary in Table 2 shows that the texts with a dummy word as an inflected verb and as a converted noun did not differ statistically with respect to their general readability, the ease of deducing the novel word’s meaning, participants confidence in deducing the meaning, and text length.

**TABLE 2:** Properties of texts introducing the novel words.

	Text condition			
	Inflected verb form		Conversion noun form	
	Mean	(*SD*)	Mean	(*SD*)
Text length (in words)	62.8	(14.8)	64.3	(14.9)
Average sentence length (in words)	15.6	(4.39)	16.2	(4.43)
Readability	5.46	(0.76)	5.19	(0.98)
Ease of deducing the meaning	5.10	(1.26)	4.88	(1.22)
Confidence in deducing the meaning	5.15	(1.22)	4.85	(1.30)

*Readability, ease of deducing the meaning and participants’ confidence were measured on 6-point Likert scales (1–6).*

In addition to the 24 texts, 6 filler texts were created that were similar to the critical texts but contained existing words only.

##### SPR Sentences

For each text, four critical sentences were created, each of them containing the novel word ending in –*en* (e.g., *BRÖSSEN*). However, in each of the sentences the novel word was used in a different function forming the four conditions of the experiment. In order to avoid orthographic cues (nouns are written with initial capitals in German), all SPR sentences were presented in capital letters (see examples below).

(1) Infinitive condition
  The novel word is used as an infinitive verb form (e.g., *sie wollen brössen*, meaning ‘they want to gawk’).
  Example:
  VIELE LEUTE WOLLEN NUR BRÖSSEN, ANSTATT SELBST ETWAS ZU TUN.
  “A lot of people just want to [gawk] instead of doing something themselves.”
(2) Inflected condition
  The novel verb was used as an inflected verb form ending in *–en* (i.e., in 3rd person plural, e.g., *sie brössen*, meaning ‘they gawk’).
  Example:
  VIELE LEUTE KOMMEN NUR UND BRÖSSEN, ANSTATT SELBST ETWAS ZU TUN.
  “A lot of people just come and [gawk] instead of doing something themselves.”
(3) Conversion noun condition
  The novel verb was used as a conversion noun in nominative or accusative case, i.e., preceded by the definite article *das* and ending in –*en* [e.g., *durch das Brössen*, meaning ‘due to (the) gawking’].
  Example:
  VIELE LEUTE KOMMEN NUR FÜR DAS BRÖSSEN, ANSTATT SELBST ETWAS ZU TUN.
  “A lot of people come just for the [gawking] instead of doing anything themselves.”
(4) Countable noun condition
  The sentence contained a concrete, countable noun in plural that was formally homonymous with the novel verb as it appeared in the text, but there was no clear meaning relationship between them (e.g., *für die vielen Brössen*, meaning ‘for the many/for all the …’). The plurality of the context was unambiguously indicated by a preceding definite or indefinite numeral requiring a plural. Note that in contrast to this countable noun condition, all conversion nouns (as in condition 3 and as introduced in the texts in Experiment L1B/L2B) are singularia tantum (i.e., they do not have any plural form) by definition. Thus, the countable noun in plural here cannot be interpreted as a conversion noun.
  Example:
  DIE LEUTE KOMMEN NUR FÜR DIE VIELEN BRÖSSEN, ANSTATT SELBST ETWAS ZU TUN.
  “A lot of people come just for all the/for the many … instead of doing anything themselves.”

As evident from the above examples, the parts of the sentences that followed the novel word were always identical in all four conditions and they were at least four words long. The part preceding the novel word that determined its word class and other grammatical properties could not be the same across all the conditions, but care was taken that there was as much overlap between the four conditions as possible.

In order to guarantee that the assumed differences in reading times are not due to reading differences that would be inherent to the four SPR sentences themselves, a pre-test was run that measured the reading times on the novel words and the words immediately following them within the sentences while no introductory texts were presented. Forty participants of the pre-test read all SPR sentences in all experimental conditions (i.e., with the novel word either as an inflected verb, an infinitive, a conversion form, or a countable noun) without any introductory texts. The participants were distributed over 4 lists such that each participant saw only one item in one of the four conditions, but each saw all four conditions equally often. None of the pre-test participants took part in the actual experiments. No differences in reading times were observed at the position of the critical word *n* (the novel word): *F*(3,895.7) = 0.29, *p* = 0.834; or the spill-over region, i.e., the following word, position *n* + 1: *F*(3,899.2) = 1.47, *p* = 0.220.^4^

The SPR sentences were related in topic to the previous text, but there was no vocabulary overlap between them and the texts except for the novel word. For each text, either none, one, or two filler SPR sentences were constructed that were also related by topic but consisted only of vocabulary typically known by the targeted learner group. The number of SPR sentences varied in order to avoid participants’ strategies and/or expectations when the new word would appear in the SPR part of the experiment and also to deflect participants’ attention away from the novel words.

For the comprehension task, a related sentence was created for each text that formed a statement that was either consistent with the meaning of the text or not. The statements referred to propositions of either the texts or the filler SPR sentences. However, they did not mention or refer to the novel word. The purpose of the task was to keep participants attentive to the texts.

#### Procedure

Prior to the experiment, participants were given written instructions, informing them that they were to read texts for comprehension and that comprehension statements would follow each text. The instructions also mentioned that the texts might contain unknown vocabulary from regional dialects or special registers, but that they were to try to grasp the text’s meaning, nonetheless. The stimuli were presented using the E-Prime 2.0 software (Psychology Software Tools, Pittsburgh, PA, United States).

Each trial consisted of three parts: reading of a text, reading of one to three SPR sentences, and assessing a comprehension statement. A trial started with the presentation of an introductory text that included the novel word or only known vocabulary (filler texts). Participants read the text silently and pressed the space bar when they were finished. When pressing the space bar, an SPR sentence written in capital letters appeared, initially with all words masked with Xs. When pressing the space bar again, the next word was revealed and the previous one was masked with Xs again (self-paced reading with a moving window, cf. Just et al., 1982). Reading times were measured. The number of SPR sentences following each text varied from one to three. One of the sentences was always the critical sentence in one of the experimental conditions.

After the presentation of the SPR sentence(s), a comprehension statement referring to the introductory text or one of the filler SPR sentences appeared on screen, and participants had to decide whether the statement was true or false by pressing one of the corresponding buttons. After the participant’s response was registered, the next trial started with an inter-stimulus interval of 1,000 ms.

Items were distributed over four experimental lists and each subject was administered to one of those lists. Each list contained all 24 texts (and 6 filler texts), but for each text only one critical SPR sentence in one of the four conditions was presented. The number of conditions was counterbalanced across lists such that each participant saw six items in each condition and that four complementary experimental lists formed a complete set. Each participant thus read each text only once followed by one of the four possible SPR conditions. Within each list, the order of trials was pseudo-randomized for each participant with fixed positions of the filler trials and the restrictions that no more than three trials with the same answer to the comprehension statement and no more than two trials with the same experimental condition followed in succession. The first trial of the experiment was always a filler trial. One session of the experiment took about 35–40 min.

### Data Preparation and Analyses

Statistical analyses for all experiments reported in the present paper were performed using linear mixed-effect models employing the software R (R Core Team, 2020). Models were fitted using the *mixed* function of package *afex* (Singmann et al., 2021). All models included random intercepts for participants and items. For all analyses, the maximal model structure was attempted (Barr et al., 2013). However, when the maximal model did not converge, the error term structure was systematically reduced using the procedure suggested by Singmann (2021). The structure of the final model is noted in the results for each analysis. For *post hoc* comparisons of contrasts of significant main effects and interactions, contrasts of estimated (marginal) means were performed using the package *emmeans* (Lenth et al., 2021) and the Bonferroni adjustment for multiple comparisons was applied for those contrasts. For the treatment of outliers, reaction time data were first winsorized with a 5% criterion, i.e., with the 2.5 and 97.5 percentile as boundaries, meaning that for each participant, all data points that fell below the 2.5th percentile or above the 97.5th percentile were set to these boundary values^5^. Additionally, and in order to compensate for non-normality of the distribution, all reaction times (in ms) were log-transformed (natural log) prior to statistical analyses. The same procedures were carried out for each of the reported experiments.

### Results

Reaction times were analyzed on the positions *n* (the novel word) and *n* + 1 (the word following the novel word; spill-over region)^6^ for the four conditions (inflected verb form, infinitive verb form, conversion noun and countable noun).

**FIGURE 1:**
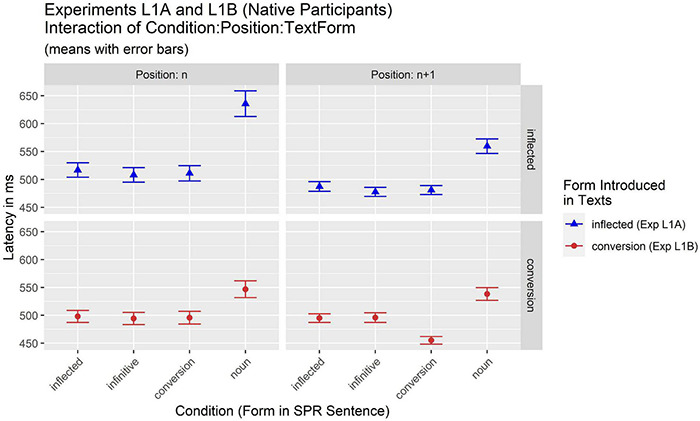
Results of Experiments L1A and L1B: native participants. (Mean latencies of critical regions in SPR sentences).

Table 3 and Figure 1 summarize the results of mean response latencies of Experiment L1A (inflected form introduced in the texts) and Experiment L1B (conversion noun introduced in texts) L1B.

**TABLE 3:** Results of Experiments L1A and L1B (mean RTs in ms and SDs).

	L1A (inflected form in texts)				L1B (conversion noun in text)			
	Position *n*		Position *n* + 1		Position *n*		Position *n* + 1	
Condition	RT	(*SD*)	RT	(*SD*)	RT	(*SD*)	RT	(*SD*)
Inflected	516.8	(267.9)	487.4	(181.1)	498.0	(222.1)	495.1	(159.5)
Infinitive	508.1	(269.9)	477.7	(168.2)	494.3	(224.4)	495.8	(178.1)
Conversion	510.9	(283.8)	481.0	(165.5)	495.6	(233.3)	455.2	(138.0)
Noun	635.8	(476.1)	559.6	(271.4)	546.8	(308.3)	538.4	(232.7)

The two L1 experiments (L1A and L1B) were analyzed together. The analysis of latencies therefore contained fixed effects for the factors Condition, Position (*n* vs. *n* + 1), and Textform (inflected form vs. conversion form introduced in the texts, i.e., experiment L1A vs. L1B). The results of the final model [log(RT) ∼ Condition * Position * Textform + (Position + Textform | Item) + (Position | Participant)] are summarized in Table 4. They reveal a main effect of Condition [*F*(3,6453.4) = 44.10, *p* < 0.001] and a significant interaction of Condition and Textform [*F*(3,6453.4) = 4.05, *p* = 0.007]. Importantly, there was also a significant higher-level 3-way interaction of Condition:Position:Textform [*F*(3,6452.0) = 2.83, *p* = 0.037] indicating that the effect of Condition was moderated by an interaction of both Position and Textform. Following this significant 3-way interaction, *post hoc* comparisons of estimated (marginal) means were computed with *p*-adjustment for the accumulated alpha error according to the Bonferroni procedure to investigate potential differences between conditions in different combinations of Position and Textform. Results (see Table 5) indicate that when the novel word was introduced as an inflected form (Experiment L1A), the pattern of results was essentially the same for positions *n* and *n* + 1: Responses to three of the four conditions were equally fast (i.e., the inflected, infinitive, and conversion condition; all *p* > 0.999), while responses for the countable noun condition were significantly slower (all *p* < 0.001). In contrast, when the novel word was introduced as a conversion form (Experiment L1B), the pattern of significant differences differed for positions *n* and *n* + 1. At position n, the results resembled the pattern also seen in Experiment L1A: there were slower responses to countable nouns compared to all other conditions. However, the effect was not so pronounced, as can be seen from the *p*-values that reveal the significance of the difference between the countable noun condition and the infinitive (*p* = 0.010) and the conversion (*p* = 0.039) condition, while there was only a marginal difference to the inflected condition (*p* = 0.081). This reduced difference is also visible in the numerical differences at position n (see also Figure 1): While in Experiment L1A there was a numerical difference of ca. 123 ms between the three faster and the slowest noun conditions, this was reduced to ca. 51 ms in Experiment L1B.

**TABLE 4:** Mixed model ANOVA table for Experiments L1A and L1B (native participants).

Effect	df	*F*	*p*-value	Signif.
Condition	3, 6453.38	44.10	<0.001	***
Position	1, 60.73	0.20	0.658	
TextForm	1, 140.25	0.19	0.665	
Condition:Position	3, 6457.96	1.19	0.314	
Condition:TextForm	3, 6453.38	4.05	0.007	**
Position:TextForm	1, 140.00	1.27	0.261	
Condition:Position:TextForm	3, 6451.96	2.83	0.037	*

Significance codes: ***p < 0.001; **p < 0.01; *p < 0.05; +p < 0.10.

**TABLE 5:** Pairwise contrasts of estimated marginal means for the predictor ‘Condition’ (by Position and TextForm) for Experiments L1A and L1B (native participants).

Contrast of condition	Textform	Position	Estimate	*SE*	df	*t*-ratio	*p*-value	Signif.
Inflected – infinitive	Inflected	*n*	0.021	0.022	6451.0	0.997	1.000	
Inflected – conversion	Inflected	*n*	0.017	0.022	6451.0	0.788	1.000	
Inflected – noun	Inflected	*n*	–0.127	0.022	6451.0	–5.892	<0.001	***
Infinitive – conversion	Inflected	*n*	–0.005	0.022	6451.0	–0.209	1.000	
Infinitive – noun	Inflected	*n*	–0.149	0.022	6451.0	–6.889	<0.001	***
Conversion – noun	Inflected	*n*	–0.144	0.022	6451.0	–6.680	<0.001	***
Inflected – infinitive	Inflected	*n* + 1	0.017	0.022	6451.0	0.812	1.000	
Inflected – conversion	Inflected	*n* + 1	0.005	0.022	6451.0	0.212	1.000	
Inflected – noun	Inflected	*n* + 1	–0.102	0.022	6451.0	–4.735	<0.001	***
Infinitive – conversion	Inflected	*n* + 1	–0.013	0.022	6451.0	–0.600	1.000	
Infinitive – noun	Inflected	*n* + 1	–0.120	0.022	6451.0	–5.547	<0.001	***
Conversion – noun	Inflected	*n* + 1	–0.107	0.022	6451.0	–4.947	<0.001	***
Inflected – infinitive	Conversion	*n*	0.015	0.022	6454.24	0.678	1.000	
Inflected – conversion	Conversion	*n*	0.005	0.022	6454.24	0.251	1.000	
Inflected – noun	Conversion	*n*	–0.054	0.022	6454.24	–2.469	0.081	+
Infinitive – conversion	Conversion	*n*	–0.009	0.022	6454.24	–0.427	1.000	
Infinitive – noun	Conversion	*n*	–0.069	0.022	6454.24	–3.148	0.010	*
Conversion – noun	Conversion	*n*	–0.059	0.022	6454.24	–2.720	0.039	*
Inflected – infinitive	Conversion	*n* + 1	0.009	0.022	6454.25	0.391	1.000	
Inflected – conversion	Conversion	*n* + 1	0.083	0.022	6454.25	3.797	0.001	**
Inflected – noun	Conversion	*n* + 1	–0.057	0.022	6454.25	–2.615	0.054	+
Infinitive – conversion	Conversion	*n* + 1	0.074	0.022	6454.25	3.406	0.004	**
Infinitive – noun	Conversion	*n* + 1	–0.066	0.022	6454.25	–3.006	0.016	*
Conversion – noun	Conversion	*n* + 1	–0.140	0.022	6454.25	–6.412	<0.001	***

p**-v**alue adjustment: Bonferroni method; degrees-of-freedom method: Satterthwaite. Significance codes: ***p < 0.001; **p < 0.01; *p < 0.05; +p < 0.10.

However, at the spill-over region (*n* + 1) a pattern emerged that is different to the so-far generally attested pattern of slower responses to countable nouns compared to (equally) faster responses to the three other conditions. While the noun condition still yielded the slowest responses, the conversion noun here elicited the fastest responses, also differing significantly from both the inflected (*p* < 0.001) and the infinitive (*p* = 0.004) condition.

To sum up, the difference between the faster conditions and the slowest (i.e., countable noun) condition was more pronounced in Experiment L1A when the novel word was introduced as an inflected word compared to Experiment L1B when the novel word was introduced as a conversion noun. In addition, an effect of faster responses to conversion nouns was observed when the novel word was introduced as a conversion noun in the text (Experiment L1B), but only at position *n* + 1.

### Discussion

With respect to the research question regarding the employment of general linguistic knowledge when establishing new lexical entries, we conclude that native speakers employ knowledge about grammar that goes beyond the information encoded in the immediate input when establishing mental representations of new words. This is indicated by the observation that participants showed no delays when presented with a form that was not in the preceding input, but whose existence could be inferred from the general knowledge about the German grammar: at position n inflected verb forms, infinitives, and conversion nouns were read equally fast regardless of the form presented in the preceding texts. At the same time, participants also showed sensitivity to the grammatical information in the input. It manifested itself with longer reading times in the countable noun condition. This form was preceded by a plural numeral in the SPR sentence, so that a noun in the plural could be predicted. However, when a word form appeared in the SPR sentence that was homonymous to the new word which participants had just acquired (via the preceding texts), but with the grammatical properties of a countable noun, participants had problems with lexical access and/or integrating this form in the sentence which resulted in the longer reading times. This also indicates that the recently established representation (based on the text input) was grammatically precise enough to be distinctly differentiated from another new representation with the same word form that participants encountered later (in the SPR sentence).

It is notable that at position n, the implausibility or surprizal effect was greater in Experiment L1A where inflected verb forms were presented in the text than in Experiment L1B where a conversion noun was presented in the text. This indicates that the word class status that was present in the input did influence the mental representation of the new word. This is further supported by the shorter reading times in the conversion condition in Experiment L1B at position *n* + 1 where the conversion noun was also presented in the input.

In the following two experiments we explored whether advanced L2 learners employ the generalized knowledge about German in the same way as the native speakers and whether their initial representations have lower resolution on the grammatical level, i.e., are more fuzzy.

## Non-Native Participants: Experiments L2A and L2B

The two experiments with non-native participants were structured and analyzed in exactly the same way as their L1 counterparts and thus only the information about the participants and the results is presented. As mentioned in the Introduction, in Czech, which was the participants’ L1, there is no analogous process to the zero-derivation found for verbs and conversion nouns in German.

### Participants

All non-native participants were native speakers of Czech who learned German as a foreign language. Their language proficiency in German was assessed prior to the actual experiments. Three different measures were obtained for each participant: a version of the Goethe Test, an online version of DiaLang (subtest on lexical knowledge), and a self-evaluation questionnaire. The classification by the three tests was not always consistent, with participants scoring at the B2 level in some test(s) and on C1 level at the other(s). Only those participants who scored at the B2 and/or C1 levels according to the Common European Framework of Reference for Languages (CEFR) in any of the three tests were selected for participation in the following experiments. As mentioned in the introduction, it can be safely assumed that Czech learners at B2/C1 level are well familiar with the investigated grammatical phenomena.

In Experiment L2A, the final group of non-native participants comprised 72 learners (62 females and 10 males) with a mean age of 23.8 years (*sd* = 7.3, range = 18–65).

In Experiment L2B, the final group of non-native participants comprised 68 learners (55 female and 13 male) with a mean age of 24.9 years (*sd* = 4.4, range = 19–41). None of the L2B participants took part in Experiment L2A.

### Results

**FIGURE 2:**
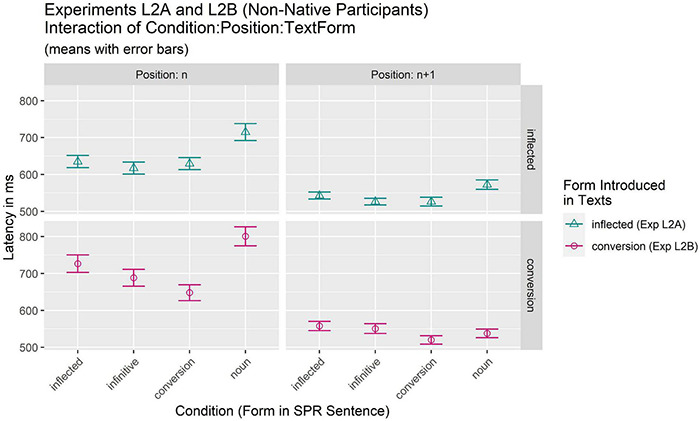
Results of Experiments L2A and L2B: non-native participants. (Mean latencies of critical regions in SPR sentences).

Results of Experiments L2A and L2B are summarized in Table 6 and Figure 2.

**TABLE 6:** Results of Experiments L2A and L2B (mean RTs in ms and SDs).

	L2A (inflected form in texts)				L2B (conversion noun in text)			
	Position *n*		Position *n* + 1		Position *n*		Position *n* + 1	
Condition	RT	(*SD*)	RT	(*SD*)	RT	(*SD*)	RT	(*SD*)
Inflected	635.0	(345.0)	543.0	(194.6)	726.6	(481.2)	557.9	(253.2)
Infinitive	617.4	(335.4)	526.6	(183.5)	688.6	(465.8)	550.6	(268.9)
Conversion	629.6	(344.1)	526.6	(244.0)	648.1	(433.1)	520.1	(229.2)
Noun	715.0	(479.0)	572.5	(266.3)	800.8	(520.2)	537.3	(237.8)

The results of the final model [log(RT) ∼ Condition * Position * Textform + (Condition + Position || Item) + (Condition + Position || Participant)] are summarized in Table 7. They reveal significant main effects of Condition [*F*(3,32.04) = 15.27, *p* < 0.001] and Position [*F*(1,108.18) = 45.87, *p* < 0.001] and a significant interaction of Condition:Position [*F*(3,6044.78) = 8.94, *p* < 0.001]. Importantly, there was also a significant 3-way interaction of Condition:Position:Textform [*F*(3,6044.78) = 5–64, *p* < 0.001] indicating that the effect of Condition was moderated by an interaction of both Position and Textform. In order to investigate potential differences between the conditions of this interaction, *post hoc* comparisons of estimated (marginal) means were computed with *p*-adjustment for the accumulated alpha error according to the Bonferroni procedure. The results of these comparisons are summarized in Table 8. For experiment L2A, in which the novel word was introduced as an inflected verb form, results for position n yielded a pattern similar to that for L1 participants: While latencies for the inflected, the infinitive, and the conversion condition were equally fast (all *p* > 0.999), they were faster than the noun condition (infinitive *p* = 0.003; conversion condition *p* = 0.020; and inflected condition *p* = 0.088). At position *n* + 1, the noun condition was slower only than the conversion condition (*p* = 0.030), while none of the other comparisons yielded significant differences (all *p* ≥ 0.230).

**TABLE 7:** Mixed model ANOVA table for Experiments L2A and L2B (non-native participants).

Effect	df	*F*	*p*-value	Signif.
Condition	3, 32.04	15.27	<0.001	***
Position	1, 108.18	45.87	<0.001	***
TextForm	1, 137.97	0.35	0.555	
Condition:Position	3, 6044.78	8.94	<0.001	***
Condition:TextForm	3, 218.16	2.50	0.060	+
Position:TextForm	1, 138.00	3.50	0.063	+
Condition:Position:TextForm	3, 6044.78	5.64	<0.001	***

Significance codes: ***p < 0.001; **p < 0.01; *p < 0.05; +p < 0.10.

**TABLE 8:** Pairwise contrasts of estimated marginal means for the predictor ‘Condition’ (by Position and TextForm) for Experiments L2A and L2B (non-native participants).

Contrast of condition	Textform	Position	Estimate	*SE*	df	*t*-ratio	*p*-value	Signif.
Inflected – infinitive	Inflected	*n*	0.029	0.025	715.6	1.175	1.000	
Inflected – conversion	Inflected	*n*	0.008	0.024	669.8	0.316	1.000	
Inflected – noun	Inflected	*n*	−0.069	0.028	118.4	−2.478	0.088	+
Infinitive – conversion	Inflected	*n*	−0.022	0.024	868.8	−0.894	1.000	
Infinitive – noun	Inflected	*n*	−0.098	0.027	128.7	−3.605	0.003	**
Conversion – noun	Inflected	*n*	−0.077	0.026	145.8	−2.986	0.020	*
Inflected – infinitive	Inflected	*n* + 1	0.027	0.025	715.6	1.077	1.000	
Inflected – conversion	Inflected	*n* + 1	0.043	0.024	669.8	1.779	0.454	
Inflected – noun	Inflected	*n* + 1	−0.030	0.028	118.4	−1.083	1.000	
Infinitive – conversion	Inflected	*n* + 1	0.016	0.024	868.8	0.683	1.000	
Infinitive – noun	Inflected	*n* + 1	−0.057	0.027	128.7	−2.089	0.232	
Conversion – noun	Inflected	*n* + 1	−0.073	0.026	145.8	−2.856	0.030	*
Inflected – infinitive	Conversion	*n*	0.062	0.026	769.8	2.437	0.090	+
Inflected – conversion	Conversion	*n*	0.115	0.025	722.4	4.596	<0.001	***
Inflected – noun	Conversion	*n*	−0.095	0.029	127.1	−3.316	0.007	**
Infinitive – conversion	Conversion	*n*	0.053	0.025	933.6	2.124	0.203	
Infinitive – noun	Conversion	*n*	−0.157	0.028	138.0	−5.617	<0.001	***
Conversion – noun	Conversion	*n*	−0.209	0.026	156.7	−7.941	<0.001	***
Inflected – infinitive	Conversion	*n* + 1	0.006	0.026	769.8	0.251	1.000	
Inflected – conversion	Conversion	*n* + 1	0.055	0.025	722.4	2.188	0.174	
Inflected – noun	Conversion	*n* + 1	0.031	0.029	127.1	1.076	1.000	
Infinitive – conversion	Conversion	*n* + 1	0.048	0.025	933.6	1.950	0.309	
Infinitive – noun	Conversion	*n* + 1	0.024	0.028	138.0	0.870	1.000	
Conversion – noun	Conversion	*n* + 1	−0.024	0.026	156.7	−0.909	1.000	

p-value adjustment: Bonferroni method; degrees-of-freedom method: Satterthwaite. Significance codes: ***p < 0.001; **p < 0.01; *p < 0.05; +p < 0.10.

A different pattern was seen when the novel word was introduced as a conversion form (Experiment L2B). While at position n the noun condition again elicited the slowest responses (all *p* ≤ 0.007), the situation for the three faster conditions was more diverse. The conversion (648.1 ms) condition was significantly faster than the inflected condition (726.6 ms) (*p* < 0.001) and the infinitive condition (688.6 ms) did not differ significantly from either of them (*p* = 0.090 and *p* = 0.203). At the same time, at position *n* + 1, no significant differences between conditions were observed.

### Discussion

The analyses of Experiments L2A and L2B reveal a different pattern of results depending on the form presented in the introductory text. Results suggest that in Experiment L2A, when the form in the introductory text was an inflected verb form, L2 learners could establish a mental representation that comprised also the conversion noun information as indicated by the verbal forms and the conversion noun having been read equally fast when presented in the SPR sentence. The L2 participants also reacted with longer reading times in the countable noun condition indicating that it was not a part of the mental representation they established for the new word. In this respect their results mirror those of the L1 participants in Experiment L1A. However, the effect was distinctly weaker at the spill-over region *n* + 1 where the countable noun differed significantly only from the conversion condition.

In Experiment L2B, however, the evidence that the L2 participants could establish a mental representation for both the verbal and the conversion noun forms when presented with the conversion form in the introductory texts is less convincing. First, results show that participants were fastest when reading the conversion form in the SPR sentence (at position *n*) indicating the superiority of this component of the new mental representation compared to the verbal component. This idea is especially supported by the observation that the inflected verb form is read significantly slower than the conversion form at position n, suggesting that the representation of the verbal component after a conversion noun in input was only weak and possibly fuzzier. Moreover, the analyses further revealed that the effect indicating processing difficulties when reading the countable noun was not present at position *n* + 1, which suggests that the established mental representation of the new form presented in the introductory text did not enable a clear differentiation between the conversion noun and another noun (countable) that should be a separate entity. This contrasts sharply with the results of the L1 experiments at both positions. Overall, the new L2 representation established after the presentation of the conversion form in the introductory text is fuzzier than the new representation established after the presentation of the verbal form in the introductory text in L2 and also fuzzier than the new representation established under the same conditions (conversion form in the introductory text) in L1. The fuzziness seems to result from the fact that when the more specific, derived conversion form is presented in the input, the L2 learners are unable to employ their general, possibly limited knowledge about German grammar so effectively as in the case when the more basic verbal form is encountered.

Finally, we investigated whether the different pattern of results obtained for native participants (Experiments L1A and L1B) and non-native speakers (Experiments L2A and L2B) could be substantiated also statistically. We therefore carried out an additional overall analysis of all four experiments containing the additional factor Language (i.e., L1 vs. L2). We were especially interested in whether the interaction of Condition:Position:Textform seen for both L1 (Experiments L1A and L1B) and L2 (Experiments L2A and L2B) participants separately was moderated by the factor Language in the overall analysis. This was indeed the case. The final model [log(RT) ∼ Condition * Position * Textform * Language + (Condition + Position + Language || Item) + (Condition + Position || Participant)] yielded a significant 4-way interaction of Condition:Position:Textform:Language [*F*(3,12273.57) = 7.67, *p* < 0.001; for full model results see Table 9].

**TABLE 9:** Mixed model ANOVA table for all 4 experiments.

Effect	df	*F*	*p*-value	Signif.
Condition	3, 39.36	18.81	<0.001	***
Position	1, 78.57	26.86	<0.001	***
TextForm	1, 278.00	0.04	0.842	
Language	1, 257.00	31.41	<0.001	***
Condition:Position	3, 12272.73	5.69	<0.001	***
Condition:TextForm	3, 438.80	4.13	0.007	**
Position:TextForm	1, 277.99	0.88	0.350	
Condition:Language	3, 439.01	1.97	0.118	
Position:Language	1, 277.99	35.82	<0.001	***
Text.Form:Language	1, 278.02	0.55	0.459	
Condition:Position:TextForm	3, 12272.73	1.06	0.364	
Condition:Position:Language	3, 12273.57	5.05	0.002	**
Condition:TextForm:Language	3, 439.01	1.46	0.225	
Position:TextForm:Language	1, 277.99	4.79	0.029	*
Condition:Position:TextForm: Language	3, 12273.57	7.68	<0.001	***

Significance codes: ***p < 0.001; **p < 0.01; *p < 0.05; +p < 0.10.

## General Discussion

In the present study, we addressed the question whether readers use previously acquired, generalized, grammatical knowledge to establish new lexical entries that would contain information not deducible from the immediate input and whether this ability depends on the properties of the word that appears in the input (i.e., verbal form vs. conversion noun form in our experiments). We further explored how these two aspects, i.e., (a) the engagement of previously acquired grammatical knowledge and (b) the specific properties of the newly encountered word interact with fuzziness as a characteristic property of not yet firmly acquired representations, typical especially for L2 learners (Bordag et al., 2021a,b; Gor et al., 2021). We took advantage of the existence of homonymous forms in the German language that can have various functions in the text and focused on the relationship between verbs and conversion nouns derived from them by a productive process. Recent evidence indicates that conversion noun information and the corresponding verbal information are represented within a joint, structured lexical entry as two distinct components (Bordag and Opitz, 2021; Opitz and Bordag, 2021).

The experiments yielded different patterns of results for native speakers and advanced L2 learners of German that partly depended on the properties of the word form presented in the introductory texts. For the L1 speakers, this factor played a minor role: they could access both the verbal and the conversion noun representational component in the SPR sentence equally fast, irrespective of which of the two forms was presented in the input (according to Hypothesis 2A). This indicates that they have good, reliable linguistic knowledge about the generalizable grammatical relations between a verb and a conversion noun and they can employ this knowledge when establishing new representations [in line with the findings of Bordag et al. (2015b)]. At the same time, their new representations of such forms have high enough resolution to be recognized and processed as different from other words that share the same surface form but have incompatible grammatical properties (homonymous countable noun forms).

Advanced L2 learners of German also possess some generalized grammatical knowledge and can employ it to a similar extent to native speakers when establishing new representations, but with specific limitations (according to Hypothesis 2B). When the form in the text input is the base (i.e., verbal) form, they can induce that the to-be-established entry needs to comprise both the verbal and the conversion noun component and their results mirror those of the L1 natives as can be seen in Experiments L2A and L1A. However, when the form in the text input is the more specific, less frequent and a derived conversion noun, the representation they establish is more incomplete and fuzzy: the verbal component is present to some degree (since the verbal conditions are still faster than the grammatically unrelated countable noun condition at least at position *n*), but it is obviously less well established than the conversion noun component as evidenced by the processing delay compared to the conversion condition. This ‘internal’ fuzziness within the lexical verb/conversion noun representation is accompanied by ‘external’ effects of fuzziness that reduces the differentiation of this representation from other, similar representations – such as the countable noun representation. It indicates that the L2 learners were able to establish a noun representation within the verb/conversion noun entry, but that this representation was not specific, clearly defined enough in its grammatical properties. In particular, the feature ‘uncountable’ or ‘singulare tantum’ which is characteristic for conversion nouns was only weakly represented in the new L2 representation. Therefore, the countable noun presented in plural contexts in the SPR sentences did not lead to pronounced, strong and lasting incongruence effects seen for L1 (cf. position *n* + 1 in Experiment L2B). This parallels the findings which the FLR hypothesis reports as evidence for fuzzy semantic and in particular phonological representations, in which an imprecise or a missing representation/encoding of a feature can lead to semantic or phonological confusions (e.g., Ota et al., 2009; Darcy et al., 2013; Cook and Gor, 2015; Cook et al., 2016; Llompart and Reinisch, 2019). Obviously, also grammatical fuzziness manifests itself through less distinct boundaries (in this case of the grammatical components of the representation), which leads to deficiencies in differentiation from neighboring representations.

All these findings are in accordance with the FLR hypothesis and the OM. While both frameworks are based primarily on the evidence from phonology, orthography and semantics, the presented study delivers evidence supporting these approaches also in the area of grammar. As suggested in the OM, morphosyntax/grammar may be another domain within the dimension of linguistic domains that comprises phonological, orthographic, and semantic domains in the model. Though the topic of fuzziness and its reduction during the ontogenetic development in the grammatical domain has not been directly addressed in previous studies, reconsideration of some of the previous findings indicates that grammar could be recognized as another domain in the model at which fuzziness operates in a similar way like in the other domains.

The OM is a model of individual lexical representations, and this is also the primary scope of the FLR hypothesis. As mentioned in the Introduction, a substantial part of grammar is handled by the mechanisms and procedures that operate on representations in the mental lexicon but may not be part of them – thus they are addressed neither by the OM nor the FLR hypothesis^7^. However, the aspects of grammar that are assumed to be stored in individual lexical entries, such as the word-class information or number information of pluralia tantum, could form the contents of the grammar or – maybe more precisely – morphosyntactic feature domain and could be grasped by the OM using its central concepts of the optimum, fuzziness, and ontogenesis.

Considering the whole grammatical domain of a single lexical entry, its optimum would be reached when all grammatical features of a given word class in the given language are acquired, including a stable representation of correctly set fixed values of the internal features. Missing, unstable or incorrectly set features would be factors that would determine the degree of fuzziness in this domain, analogically to how fuzziness is captured in the FLR hypothesis for the other domains. From a more differentiated perspective, ontogenetic development of the individual features toward their optima can be considered, too. As an example, research on grammatical gender (e.g., Bordag and Pechmann, 2007) indicates that during its ontogenesis, a fuzzy phase of gender value computation based on various sources (phonological form of the word, its L1 gender value, unstably set L2 gender value) precedes the final, optimum, stage when the gender value is firmly fixed and automatically retrieved (not computed each time anew). Similarly, the results of the present study suggest that the examined newly established L2 representations follow a developmental trajectory from a weak representation of a word class information that leads to processing difficulties when accessing the verbal component of the verb/conversion noun representation and from low-resolution representation of the fixed number that compromises the differentiation from homonymous, but separate countable noun representations (positions *n* + 1 of Experiments 2A and 2B) toward a more precise grammatical representation that manifests itself in functional equivalence comparable to the L1.

Though the present study delivers promising results in areas such as incidental vocabulary acquisition, grammar acquisition, and research on FLR and ontogenetic development of individual representations at the grammar domain, more research is clearly needed to substantiate the presented claims. As an example, the current study was limited in that we explored advanced L2 learners and hypothesized about the ontogenetic development of their newly established representations based on the comparison with L1 and on acquisition in two differently difficult learning contexts (experimental versions A and B). In order to gain a clearer picture of, for instance, such developmental aspects, future research should address comparisons between participants at different proficiency levels and in longitudinal studies. Moreover, examining different L1–L2 pairings, for example, could help determine the role of cross-linguistic transfer in resolving fuzziness. With respect to practical aspects of language instructions, it would also be interesting to explore whether fuzziness in the explored area can be reduced by particular teaching methods or training.

## Data Availability Statement

The raw data supporting the conclusions of this article and the R script used to generate all reported results are publicly available in the Mendeley database: doi: 10.17632/548vjgyk6t.1.

## Ethics Statement

The studies involving human participants were reviewed and approved by German Research Council (DFG). The patients/participants provided their written informed consent to participate in this study.

## Author Contributions

All authors listed have made a substantial, direct, and intellectual contribution to the work, and approved it for publication.

## Conflict of Interest

The authors declare that the research was conducted in the absence of any commercial or financial relationships that could be construed as a potential conflict of interest.

## Publisher’s Note

All claims expressed in this article are solely those of the authors and do not necessarily represent those of their affiliated organizations, or those of the publisher, the editors and the reviewers. Any product that may be evaluated in this article, or claim that may be made by its manufacturer, is not guaranteed or endorsed by the publisher.

## Footnotes

1 Clahsen and Felser (2017) explicitly classify their Shallow Structure Hypothesis as a multiple-pathways model that assumes at least two processing routes that operate in parallel. One of the routes involves creating detailed syntactic structures; the other one is syntactically shallower (i.e., characterized by deficiencies in hierarchical syntactic organization). Learners also have access to both paths in principle in their L2, but they have to rely much more on the shallower one.

2 These difficulties are primarily accounted for by two factors: (1) Later learning onset in L2 that is associated with lower learning outcomes (e.g., DeKeyser, 2012; Hartshorne et al., 2018; Bylund et al., 2021) and (2) the L1 mental lexicon and phonological system already being in place when L2 lexical learning starts. L2 learners thus need to overcome the influence of L1 in developing the L2 system (e.g., Jarvis, 2000; Barrios and Hayes-Harb, 2020, 2021; Llompart, 2021).

3 The two words were presented in two steps; participants made a grammaticality judgment over the whole phrase.

4 Statistical analyses were conducted in parallel to those reported in detail for the experiment data (i.e., linear mixed effects models with log-transformed reaction times etc., for details see below).

5 This follows recommendations of data treatment of L2 reaction times (Nicklin and Plonsky, 2020).

6 Exploratively, we also looked at later spill-over regions, i.e., positions *n* + 2 and *n* + 3. However, at these positions the effects observed on *n* and *n* + 1 were already receding and mostly not significant. We thus analyzed and report only the *n* and *n* + 1 positions.

7 Though the FLR hypothesis discusses only representational fuzziness (which is also in the focus of our present study), it acknowledges the existence of fuzziness at the processing level, too (Gor et al., 2021).
